# Influence of Post-Heating Treatment on the Sensory and Textural Properties of Stirred Fermented Milk

**DOI:** 10.3390/foods12163042

**Published:** 2023-08-13

**Authors:** Fei Gao, Dongdong Li, Hongliang Li, Han Chen, Xueying Mao, Pengjie Wang

**Affiliations:** 1 College of Food Science & Nutritional Engineering, China Agricultural University, Beijing 100083, China; tb2022306009@cau.edu.cn; 2 Beijing Advanced Innovation Center for Food Nutrition and Human Health, Department of Nutrition and Health, China Agricultural University, Beijing 100190, China; lidongdong53@mengniu.cn (D.L.); h.chen@cau.edu.cn (H.C.); 3 Inner Mongolia Mengniu Dairy (Group) Co., Ltd., Hohhot 011500, China; lihongliang@mengniu.cn

**Keywords:** yogurt, graininess, texture, rheology, tribology, multiple linear regression

## Abstract

The purpose of this study was to investigate the post-heating induced changes in the textural and sensory characteristics of stirred fermented milk. The textural and rheological properties of post-heating fermented milk (55–85 °C, 25 s) with respect to viscoelastic behaviors, viscosity, textural parameters, etc., were monitored, and the friction behaviors and sensory attributes were assessed. Treatments below 65 °C/25 s increased the textural properties of fermented milk such as gel strength, firmness, and viscosity, due to the moderate aggregation and increased linkages of microgels. In this case, despite the size and amount their aggregates increased (~15–~21 μm), they exhibited similar frictional behaviors and sensory attributes. However, treatments above 65 °C/25 s degraded textural properties due to excessive aggregation (~46–~63 μm), accompanied by unacceptable grainy attributes, which could be characterized by their good correlations with tribological coefficients and particle size parameters. These findings could provide an understanding of the quality formation of post-heating fermented milk and a perspective to improve the textural defects of ambient fermented milk products.

## 1. Introduction

Traditionally, yogurt is a chilled snack typically served at the breakfast table, but ambient yogurt overturns this convention mode. For millions of Chinese people, particularly young people, ambient yogurt is now a drink consumed in portion packs while on the go: a trend that is spreading across Asia, Africa, and Latin America. Ambient yogurt has a long shelf-life of 3–6 months at room temperature, and it can provide a nutritional benefit similar to traditional fermented chilled yogurt except for viable probiotic bacteria, and since it does not require refrigeration, it can also be consumed on the move. Growing consumer demand for convenience, nutritional value, and taste is driving the popularity of ambient yogurt in China and beyond.

Although ambient yogurt has broad market potential, it has texture defects in terms of roughness (irregularities in the yogurt matrix) and graininess (powdery sensation), which have always been some of the most prominent quality problems reported by consumers [1]. Compared with chilled yogurt products, ambient yogurt products generally require additional thickeners, emulsifiers, and stabilizers to maintain system stability and improve the sensory attributes of yogurt products such as smoothness, consistency, viscosity, and appearance [2]. However, the additional use of these additives not only increases the production costs, but also is not conducive to clean labels, which are not expected by consumers and dairy manufacturers. The post-heating operation is the direct cause of texture defects in ambient fermented milk products [3], and the post-heating temperature determines the extent of the related properties of these texture defects [4]. A higher post-heating temperature or longer holding time generally tends to result in a rougher and more grainy structure of fermented milk [5], accompanied by changes in the system’s textural properties such as viscoelastic behaviors, and sensory properties such as graininess [3].

Temperature is an important process parameter for producing ambient fermented milk products with these common textural defects regarding roughness and graininess [3] because the increasing temperature changes the balance between hydrophobic interaction and electrostatic repulsion, increases thermal motion of the particles, and promotes extensive particle rearrangement, leading to the formation of dense clusters of aggregated particles [3]. The aggregation of particles and increase of particle size indicate the transition of the gel from a compact structure to larger aggregates [6], in which case, the strength and number of each type of bond intra- or inter-particles contained in the particle unit constituting the aggregates contribute to the overall character of the final gel [5]. Therefore, the strength of the gel and its flow behaviors (viscoelasticity, thixotropy, etc.) are first and foremost affected by heat treatment. Changes in these textural properties of fermented milk collectively contribute to the complex perception of sensory attributes regarding roughness and graininess. In previous studies, the particle characteristics and textural properties of fermented concentrated milk products, such as fresh cheeses (Quark-type products) [3] and fat-free high-protein yogurt (concentrated/strained Greek-style yogurt) [6], were investigated based on different post-heating conditions. The purpose was to prevent excessive acidification, improve the texture of the products after packaging, and extend the shelf life [6]. The post-heating treatment (relatively low temperature combined with a long time, 23–54 °C/1–300 min) was shown to have a regulatory effect on the particle growth behavior of concentrated fermented milk (protein content 8.2% (*w*/*w*)) [3]. The increased temperature (23–54 °C/1 min or 300 min) and extended holding time (23 °C, 38 °C, 54 °C/1–300 min) enhanced both extensive particle rearrangement during gel formation and the formation of dense clusters of aggregated particles [3], resulting in a grainy texture in fresh cheese products. Furthermore, the shelf-life stability of Quark-type products after whey separation can be greatly influenced by the post-heating treatment (relatively high temperature combined with short time, 55–75 °C/30–60 s), which was manifested in particular by the prevention of off-flavor development such as bitterness, stale, or acidic; the cessation of lactic acid production; and the reduction of the proteolytic activity contributed by rennet and starter bacterial enzymes [7]. Moreover, if the Quark-type products were post-heating (75 °C/2–3 min) treated in the presence of undenatured whey protein, the firmness of these rather soft fresh cheeses can also be effectively increased after acid coagulation and whey removal [7]. On the other hand, it has been reported that for Quarks (up to 40% (*w*/*w*) fat-in-dry matter, FDM) without any stabilizer added, the limit condition for the post-heating treatment is pH < 4.2 and temperature < 60 °C. Otherwise, the protein coagulum would cause the product texture to become too hard, rough, and accompanied by sandy/grainy defects [7]. This problem also particularly occurs in low-fat cheeses with FDM less than 10% and concentrated yogurts [7]. Overall, the post-heating treatment has a significant effect on the particle characteristics and texture properties of fermented milk products, which not only depend on the heating temperature and holding time, but also on the component properties of the milk, mainly protein and fat content.

However, to the best of our knowledge, there are currently no experimental studies discussing the impact of post-heating for a relatively high temperature combined with a short time on the sensory and textural properties of stirred fermented milk, particularly focusing on the development of graininess caused by extensive particle aggregation and rearrangement. The post-heating treatment below 85 °C/25 s is a commonly applied secondary pasteurization parameter in the production of ambient yogurt products, which is a comprehensive consideration based on the quality loss and shelf-life extension of these products in the industry. Based on this, this article was designed to investigate the changes in the texture and rheology of fermented milk, including the viscosity, texture, viscoelasticity, and thixotropy, etc., with an increasing post-heating temperature from 55 °C to 85 °C for a fixed holding time (25 s). The main purpose was to reveal the reasons for these changes in textural and rheological properties from the particle aggregation process reflecting in particle sizes throughout the post-heating treatment process, and how their changes further affected the sensory attributes of fermented milk, especially graininess. We expect this study to provide a preliminary understanding for the development of the sensory and textural properties of fermented milk, and to provide an intervening perspective for improving the sensory and textural defects of ambient yogurt by controlling aggregation.

## 2. Materials and Methods

### 2.1. Sample Preparation and Processing

#### 2.1.1. Stirred Fermented Milk

Ultra-high temperature-treated milk (3.2% protein, 4.0% fat) (Inner Mongolia Mengniu Dairy (Group) Co., Ltd., Hohhot, China) was used as the base to heat up 45 °C to 50 °C in the fermentation tank (SY-PA-04-04, Shanghai Shunyi Technology Co., Ltd., Shanghai, China). Granule sugar with 6.0% (*w*/*w*) and low-ester pectin (CP Kelco Co., Ltd., Shanghai, China) with 0.30% (*w*/*w*) were added and stirred with 550 rpm using an electronic stirrer (R30A, FLUKO (Shanghai) Technology Development Co., Ltd., Shanghai, China) until they were completely and evenly mixed with the milk base. Subsequently, continuing to heat up, the mixture was brought to 60 °C, and the above mixed system was homogenized at a pressure of 40 bar/150 bar (APV-2000, SPX (Shanghai) Flow Technology Co., Ltd., Shanghai, China). After homogenization, the mixed system was continuously heated up to 85 °C and kept for 20 min, and then cooled down to 42 °C by flowing water for inoculation and fermentation (0.03% (*w*/*w*)), *Streptococcus thermophilus* (MN-ZLW-002, China General Microbiological Culture Collection Center (CGMCC), CGMCCNO.3817). When the pH of the fermented milk dropped to 4.50 ± 0.04 (about 5–6 h), the fermentation was terminated. Then, the stirring operation was performed at 550 rpm (R30A, FLUKO (Shanghai) Technology Development Co., Ltd., Shanghai, China) to disrupt the gel system.

#### 2.1.2. Post-Heating Treatments

The stirred fermented milk was divided into five parts, with one part left untreated (NT group), and the remaining four parts were batch heated in the pasteurized fermentation tank (SY-PA-04-04, Shanghai Shunyi Technology Co., Ltd., Shanghai, China) to a central temperature of 55 °C, 65 °C, 75 °C, and 85 °C, respectively, and then kept for 25 s, and then batch cooled down to a central temperature of 25 °C using circulating water in another pasteurized fermentation tank (SY-PA-04-04, Shanghai Shunyi Technology Co., Ltd., Shanghai, China), and then stored overnight at 4 °C along with the NT group.

In particular, the samples required for the post-heating treatment in each treatment group were divided into fermenters (samples with a volume of 1.5 L from each treatment group were loaded into fermenters with a volume of 2.0 L), and the time required for these samples to be heated from 42 °C to a center temperature of 55 °C, 65 °C, 75 °C, and 85 °C in a pasteurization tank at 95 °C was 19 ± 1.41 s, 48 ± 7.07 s, 82 ± 15.56 s, and 204 ± 33.94 s, respectively. Furthermore, all stirring operations involved in sample preparation were performed at 550 rpm using an electric stirrer (R30A, FLUKO (Shanghai) Technology Development Co., Ltd., Shanghai, China), in order to avoid any system differences caused by shearing rate differences. At the same time, the addition of low-ester pectin was selected as 0.30% based on the heating stability of fermented milk according to the preliminary experiments.

### 2.2. Particle Size Distribution

Before the measurement, according to the sample preparation method in Section 2.1, additional fermented milk samples of the five treatment groups (pH = 4.50 ± 0.07) were prepared in order to extract the whey of the corresponding group samples by centrifugation (5000× *g* × 30 min) at 4 °C. The extracted whey was filtered using an ultrafiltration membrane (cut-off 10,000 Da) and then heated to 37 °C in the water bath, and subsequently used to disperse the corresponding samples during particle size measurement. The dispersant refractive index was set at 1.32, and the particle refractive index was set at 1.46. The particle size distributions of all samples were determined by a laser diffraction instrument (Malvern Mastersizer S, Malvern Instruments, Worcestershire, UK) at 37 ± 1 °C. The particle size result for each sample was obtained from the average of 6 consecutive measurements. Since the particle size distributions of the samples from all treatment groups were unimodal, the volume-weighted mean diameter D_[4,3]_ was used to represent particle sizes.

### 2.3. Frequency Sweep, Strain Sweep, and Apparent Viscosity

#### 2.3.1. Frequency Sweep

Dynamic rheological measurements were carried out in a stress-controlled rheometer (MCR302, Anton paar GmbH, Graz, Austria) using a 1 mm gap parallel-plate sensor. The fermented milk sample was placed on the rheometer’s bottom plate. The top plate was gradually lowered until the gap was 1 mm and a cone (50 mm diameter; 1° angle) was used. The plate temperature was kept constant at 37 °C. Strain sweep tests were performed from 0.01% to 50% at frequency 1 Hz to ensure that viscoelastic measurements were performed in the linear viscoelastic region (LVR). Following that, samples were subjected to a frequency sweep from 0.01 Hz to 10 Hz in the LVR at a constant shear strain of 0.5%. The elastic modulus (G′) and viscous modulus (G″) were measured as a function of frequency.

#### 2.3.2. Strain Sweep

The strain sweep procedure was the same as the frequency sweep, and specific parameters were set as follows: the applied strain was 0.01–1000%, angular frequency (ω) was 10 rad/s, and the plate temperature was kept constant at 37 °C.

#### 2.3.3. Apparent Viscosity

The shear stress was recorded as shear rates increased from 0 to 100 s^−1^ at 37 °C.

### 2.4. Thixotropic Properties

The thixotropic properties of the samples were measured at 37 °C using a Rheolab QC rotational shear rheometer equipped with a conical CC 27 probe with a diameter of 20 mm (Rheolab QC, Anton paar GmbH, Graz, Austria). The shear rate varied from 0 to 100 s^−1^ for the upward curve and from 100 to 0 s^−1^ for the downward curve, and the thixotropic behavior of fermented milk was evaluated by calculating the thixotropic loop area between the upward and downward flow curves.

### 2.5. Texture Properties

Textural attributes of the samples including firmness, consistency, and cohesiveness were analyzed by a single compression test with a TA.XT Plus Texture Analyzer (Stable Micro System, Godalming, Surrey, UK) equipped with a back extrusion cell disc (A/BE; diameter 40 mm; distance 30 mm). The speed before, during, and after the test was 1.00 mm/s, and the trigger force was Auto–10.0 g.

### 2.6. Tribology

Tribological measurements were performed on a stress-controlled rheometer (model DHR-1, Discovery Hybrid Rheometer; TA Instruments, New Castle, DE, USA), equipped with ring-on-plate tribo-rheometry. According to the method described by Luo et al. [8], the plate was covered with a rough plastic surface of 3M Transpore Surgical Tape 1527-2 (3M Health Care, Brookings, SD, USA). After each measurement, the tape was replaced and the tribo-rheometry was cleaned and dried. A small amount of the sample (approximately 2 mm thick) was gently spread to cover the surface of the lower plate, and the samples were equilibrated at 37 °C for 30 s before measurement, and then measured at 37 °C with a constant normal force of 2 N. The friction coefficients were measured for rotational speeds ranging from 0.15 to 300 mm/s.

### 2.7. Sensory Analysis

Sensory evaluations including a quantitative descriptive analysis (QDA) and consumer preference analysis (CPA) by 10 trained panelists (10 females, mean age: 34 y) and 50 consumers (32 females, 18 males, mean age: 27 y) were performed in separate booths in the sensory laboratory of the Ambient Dairy R&D Center of Mengniu Dairy Group. In particular, 10 female trained panelists were all from Inner Mongolia of China and had at least 3 years of professional experience in the sensory evaluation of dairy products. Their age distribution ranged from 25 to 45, with 3 panelists aged between 25 and 30, 6 panelists aged between 31 and 40, and 1 panelist aged between 41 and 45. Among these panelists, 9 had undergraduate experience and 1 had master’s experience. In the questionnaire survey of daily yogurt consumption over the past three months, there were 8 panelists who consumed yogurt 4–6 times a week, 2 panelists who consumed yogurt 2–3 times a week, and no panelists who consumed yogurt once a week or less. Moreover, they also self-reported to be the main shoppers in their households, frequently purchasing and consuming yogurt because they believed that maintaining a regular yogurt consumption habit was beneficial to their health. Meanwhile, the randomly selected 50 consumers also had some experience in yogurt evaluation and consumption habits (at least once a week in the past three months), and they were familiar with sensory definitions related to yogurt textural properties. The focus of this work was on the acceptability of graininess for CPA and other associated textural attributes of post-heating fermented milk samples, including five attributes: thickness (resistance to flow in the mouth before saliva modifies the sample), smoothness (perceived smoothness of the sample squeezed between palate and tongue), graininess (perceivable amount of small particles causing a powdery sensation), stickiness (degree to which the sample sticks to the teeth and palate), and residual coating (intensity of residues left in the mouth after swallowing) for QDA.

An assessment with respect to attribute intensity was referenced to the generalized Labeled Magnitude Scale (gLM) [9] and slightly modified, in which 0 point was defined as “imperceptible”, 1 point as “extremely weak”, 2 points as “very weak”, 3 points as “weak”, 4 points as “somewhat weak”, 5 points as “moderate (neither strong nor weak)”, 6 points as “somewhat strong”, 7 points as “strong”, 8 points as “very strong”, 9 points as “extremely strong”, and 10 points as “the strongest imaginable sensation of any kind”. The assessment with respect to graininess acceptability was referred to using the Labeled Affective Magnitude Scale (LAM) [10], which was slightly modified, in which 0 point was defined as “greatest imaginable disliked”, 1 point defined as “extremely disliked”, 2 points defined as “very dislike”, 3 points defined as “dislike”, 4 points defined as “somewhat disliked”, 5 points defined as “moderate (neither like nor dislike)”, 6 points defined as “somewhat liked”, 7 points defined as “liked”, 8 points defined as “very liked”, 9 points defined as “extremely liked”, and 10 points defined as “greatest imaginable liked”. Before the sensory evaluation, all samples were transferred from refrigerated conditions at 4 °C, and about 60 g of each fermented milk sample was placed in a 100 mL odorless transparent plastic cup coded with a 3-digit random number, and equilibrated at room temperature for 30 min. During sensory testing, palate-cleansing water was prepared at intervals between samples to prevent cross-effects between samples.

### 2.8. Statistical Analysis

All experiments except the sensory analysis were carried out in triplicate and the results were expressed as the mean ± standard deviation. A statistical analysis of all data in this paper was conducted using IBM SPSS Statistical 26.0 (SPSS Inc., Chicago, IL, USA), Origin 9.0 software (Originlab Corporation, Inc., Massachusetts, USA), and Unscrambler 10.4 software (CAMO, Trondheim, Norway). Data normality was determined using the Shapiro–Wilk’s test and then the homogeneity of variance was analyzed using the Levene’s test. For the data that were normally distributed, a one-way analysis of variance (ANOVA), followed by Fisher’s LSD test (homoscedasticity) or Dunnett T3 test, were used to describe the means with 95% confidence. For non-normally distributed data, a non-parametric Kruskal–Wallis 1-one ANOVA, followed by a Dunn test for multiple comparisons, were applied. *p* ≤ 0.05 represents a significant difference. The relationships between the instrument and sensory data were investigated using the PCA, with sensory attributes as dependent Y-variables and instrument parameters as independent X-variables. Regression models of sensory attributes, with a focus on graininess and graininess acceptability, were established by stepwise multiple linear regression. *p*-values for taking a variable into the model were set to 0.05 and to 0.1 for removing it from the model.

## 3. Results and Discussion

### 3.1. Particle Size Distribution of Post-Heating Fermented Milk

**Figure 1:**
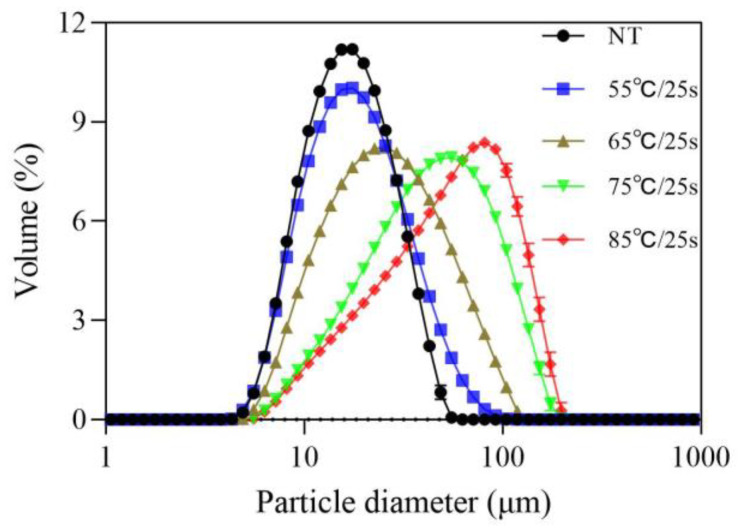
Particle size distribution of stirred fermented milk with different post-heating temperature.

The PSD curves obtained for fermented milks with different post-heating treatments shown in Figure 1 appeared to be unimodal, with the peak width of the curve increasing with the post-heating temperature and moving in the direction of the larger particle size.

The PSDs were well-summarized by D_[10]_, D_[50]_, and D_[90]_ percentiles as well as the volume mean particle size (D_[4,3]_), reported in Table 1.

It can be observed that an increase in post-heating temperature had a significant positive effect on particle size. All particle size parameters, including D_[10]_, D_[50]_, D_[90]_, and D_[4,3]_, increased significantly (*p* ≤ 0.05) with the increase in the heat treatment temperature. According to Fidaleo et al. [11], Gilbert et al. [12], and Hahn et al. [13], D_[90]_ and D_[4,3]_ values were selected as representative parameters reflecting the change of particle size in this study because they had been shown to reveal the behavior of large particle clusters responsible for grainy defects [3,4] and to explain the perceived graininess by a trained panel [11]. Obviously, these two parameters were significantly increased in the treatment groups above 65 °C/25 s compared to in the treatment groups below 65 °C/25 s (*p* ≤ 0.05).

In previous studies, little attention had been paid to the influences of post-heating temperature above 55 °C on stirred yogurt in experimental articles, but it was certain that the post-heating temperatures had an important impact on the function, structure, and size of yogurt microgels [5,12]. According to previous reports, in the case of other post-processing conditions after fermentation, the post-heating temperature altered the density or morphology of yogurt microgels, which in turn affected their reorganization and size during post-aggregation [4,5,12,13]. This phenomenon was usually accompanied by a decrease in the number of particles and an increase in particle size, with an increase in large particle clusters at the expense of a drop in smaller ones [3]. More specifically, smaller casein-based building blocks underwent further fusion into larger clusters of aggregated particles [3]. Furthermore, the microstructure of yogurt gel was also affected [14]. Low-temperature treatment at 20 °C produced skim stirred yogurt with small microgel particles, while high-temperature treatment at 42 °C before storage produced heterogeneous gels with large microgel particles.

### 3.2. Flow Behavior and Textural Properties of Post-Heating Fermented Milk

#### 3.2.1. Frequency Sweep and Strain Sweep

**Figure 2:**
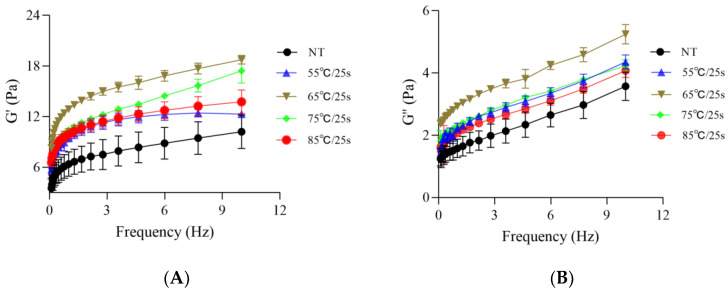
Storage modulus values (G′, (**A**)) and loss modulus values (G″, (**B**)) of post-heating stirred fermented milk.

Frequency sweep is a non-destructive method for evaluating the viscoelasticity of yogurt. As shown in Figure 2A,B, the storage modulus (G′) and loss modulus (G″) of fermented milk samples were frequency-dependent. All fermented milk samples exhibited viscoelastic features, with G′ being higher than G″ across the frequency range. G′ and G″ represent the energy stored and dissipated per oscillation cycle, respectively, indicating the strength and number of bonds in the network, while also reflecting the contribution of stronger and weaker protein–protein interactions, respectively [13]. As depicted in Figure 2, an increase in the post-heating temperature (55 °C/25 s to 65 °C/25 s) led to an increase in the storage modulus G′ (Figure 2A) and loss modulus G″ (Figure 2B) of the samples, which reflected an increase in the energy stored in each oscillatory cycle as a result of higher post-heating temperatures, which may point to stronger and more frequent protein–protein interactions, at least for some structural elements [13]. Meanwhile, the energy dissipated during each oscillation cycle also increased substantially at each temperature, which would point to weaker or fewer protein–protein bonds per cross-section [13]. Interestingly, when the post-heating temperature exceeded 65 °C/25 s, a decrease in the values of G′ and G″ was observed in the more intensely heat-treated samples. This result may be explained by the impact of post-heating temperature on the development of microgels and the degree of structural rearrangement at different structural levels. According to Mokoonlall et al. [15], the rearrangement of gel particles at four structural levels had an effect on the rheological properties of gel systems, i.e., (i) elementary particles at the molecular level such as caseins, (ii) particle level consisting of several elementary particles, (iii) cluster level involving the particle strands, and (iv) macroscopic gel level. In general, the first two rearrangement types led to the formation of larger aggregates [15], resulting in an increase in the gel storage modulus, while more intense temperatures (75 °C/25 s and 85 °C/25 s applied in this paper) may have contributed to greater disruption of the electrostatic forces between protein particles [16], resulting in a weaker linkage between particle clusters and inhibiting the successive formation of a dense gel network. Additionally, more intense heating treatments drove the aggregation of larger clusters with more particles through increased hydrophobic interactions [6] and led to a rough, less shear-resistant gel structure consisting of larger irregular microgels. Therefore, the fragility and heterogeneity of the gel structure at higher temperatures may explain the corresponding decreases in the rheological characteristics of those samples under such conditions.

**Figure 3:**
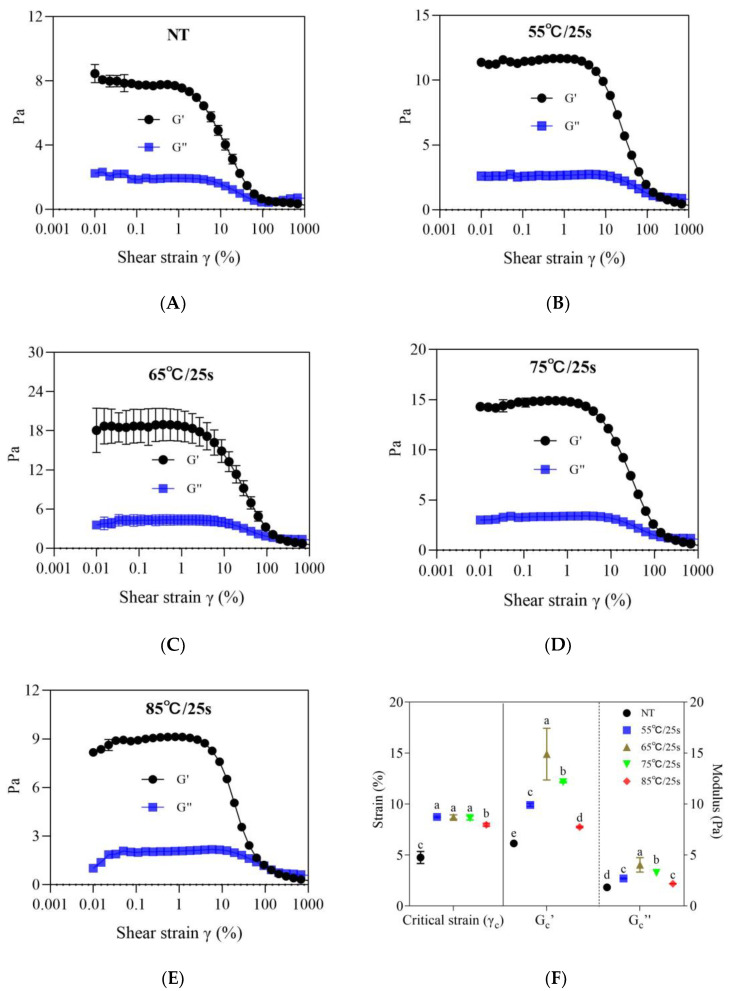
Amplitude sweep curves of post-heating stirred fermented milk. Note: The error bars represent the standard error of the means. (**A**–**E**) represent the NT group, 55 °C/25 s group, 65 °C/25 s group, 75 °C/25 s group, and 85 °C/25 s group, respectively, and (**F**) represents the critical strains of all groups and their corresponding changes in elastic modulus (G_c_′) and loss modulus (G_c_″). Values with different letters, in (**F**), are significantly different (*p* ≤ 0.05).

Strain sweep provided the variation of G′ and G″ of these samples with increasing strains, and the higher values of both determined the stability of the samples; that is, when G′ < G″, the viscous deformation behavior of the samples was superior to elastic deformation behavior, which exhibited a fluid characteristic. Conversely, when G′ > G″, the elastic deformation behavior of the samples dominated, and their structure exhibited certain rigidity and solid properties [17]. In addition, the linear viscoelastic region of the samples can also be obtained by strain scanning, which indicated the maximum strain or stress that the samples can withstand without affecting their own structure [18]. As shown in Figure 3A–E, no significant changes in G′ and G″ of the samples were observed in the respective linear viscoelastic regions, and G′ > G″, which indicated that their gel network structure was not destroyed in this strain range and showed relatively strong elasticity and solid properties [19]. As the applied strain was further increased to reach or exceed the critical strain (γ_c_) of the system, the modulus (G′ and G″) began to decrease gradually, indicating that the network structure of the system was disrupted, while the fermented milk began to flow, showing fluid properties. The values of G_c_′ and G_c_″ at critical strain and the critical strain value (γ_c_) of fermented milk samples are shown in Figure 3F. It can be observed that the corresponding G_c_′ and G_c_″ values of the samples increased significantly (*p* ≤ 0.05) with the post-heating temperature increasing from 55 °C/25 s to 65 °C/25 s, and then decreased significantly (*p* ≤ 0.05) when the temperature exceeded 65 °C/25 s. Meanwhile, a significantly lower γ_c_ value (*p* ≤ 0.05) was observed in the 85 °C/25 s group than that in the other treatment groups.

The changes in the values of γ_c_, G_c_′, and G_c_″ of the samples reflected the strength of their intrinsic gel structure stability [5], and the increase in these parameters was more likely due to the reconstruction of the protein network in fermented milk after post-heating treatments. Furthermore, the decrease in these parameters was considered to be associated with the formation of a rough, less shear-resistant gel structure consisting of larger irregular microgels under excessive post-heating conditions (Section 3.1).

#### 3.2.2. Thixotropic Properties

**Figure 4:**
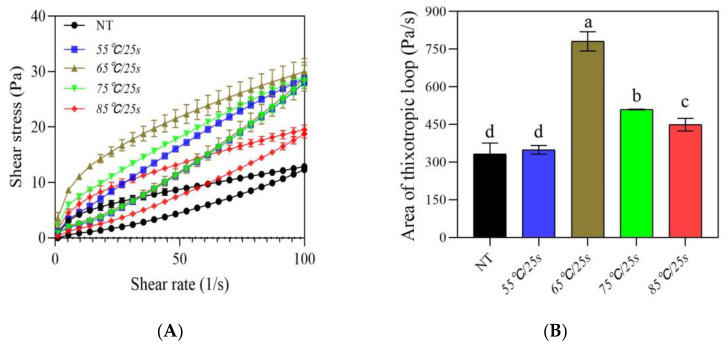
Thixotropic properties (**A**) and area of thixotropic loop (**B**) of post-heating stirred fermented milk. Note: The error bars represent the standard error of the means. Values with different letters, in (**B**), are significantly different (*p* ≤ 0.05).

As depicted in Figure 4, when the samples were sheared at increasing and then at decreasing shear rates, the shear stress first increased to a maximum value, and then decreased, showing shear thinning and thixotropic behavior, i.e., an irreversible change in the structure with time under shear [13]. The enclosed area between the up-and-down curves was often used as a measure of the degree of structural breakdown during the shearing cycle, and the larger the area, the higher the thixotropic effect [20]. As shown in Figure 4B, the thixotropic loop area of the fermented milk samples gradually increased with the increase in the post-heating temperature from 55 °C/25 s to 65 °C/25 s (*p* ≤ 0.05), and then decreased with the increase in the temperature from 75 °C/25 s (*p* ≤ 0.05) to 85 °C/25 s (*p* ≤ 0.05). These findings suggested that sample structure destruction below 65 °C/25 s required a higher shear stress (which corresponded to a larger shear stress in the shearing rate stage of 0–100 s^−1^) and was more difficult to recover after being destroyed, while the structural behavior of samples above 65 °C/25 s was the opposite. These findings were in accordance with trends in the storage modulus and critical strain (γ_c_, Figure 3F) in the frequency sweep (G′, Figure 2A) and strain sweep (G_c_′, Figure 3A–E). The development of thixotropy in yogurt was associated with particle breakage due to the disruption of weak forces such as ionic bonds, van der Waals forces, and hydrophobic interactions between protein particles [21]. Hahn et al. [13] reported a positive correlation between storage modulus and shear stress due to the contribution of the strength and number of bonds in the network to the interaction between protein particles/clusters. Miocinovic et al. [22] stated that critical strain was a measure of the difficulty of breaking strands, representing the fracture properties of the gel that depended on the number of bonds per cross-section of a strand as well as on the strength of each bond, and a lower critical strain value of the fermented milk gel usually indicated that its structure was more susceptible to rearrangement and fracture. Based on these shared structural foundations, the storage modulus, critical strain, and thixotropy trends of the samples in this paper showed a certain consistency.

#### 3.2.3. Apparent Viscosity

**Figure 5:**
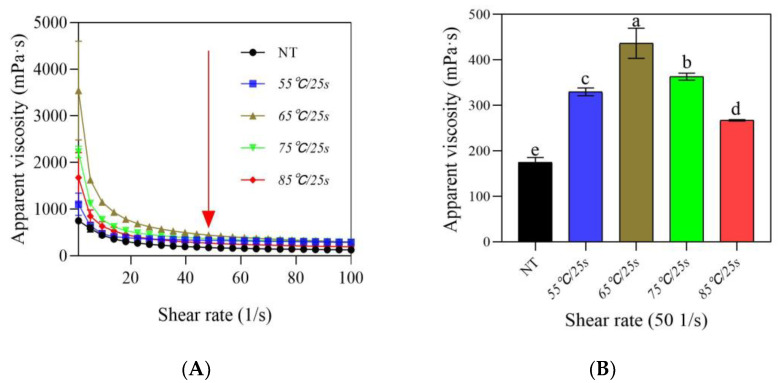
Changes in apparent viscosity of post-heating stirred fermented milk at 0–100 s^−1^ (**A**) and 50 s^−1^ (**B**). Note: The error bars represent the standard error of the means. Values with different letters, in (**B**), are significantly different (*p* ≤ 0.05).

The flow characteristics of yogurt are closely related to its intrinsic characteristics such as mean particle size and dynamic stability [14]. As shown in Figure 5A, for all fermented milk samples, the apparent viscosity decreased with the increase of the shear rate, showing typical pseudoplastic behavior. It has been reported that the viscosity at a shear rate of 50 s^−1^ correlated well with the perceived thickness, stickiness, and sliminess of various foods from Newtonian fluids to thick emulsions [23]. As can be seen from Figure 5B, the apparent viscosity at η_50_ of samples first increased significantly (*p* ≤ 0.05) as the post-heating temperature increased from 55 °C/25 s to 65 °C/25 s, and then decreased significantly (*p* ≤ 0.05) as the temperature increased from 75 °C/25 s to 85 °C/25 s, which indicated that the post-heating temperature produced a significant influence on the viscosity of stirred gels. The microgel size was considered to be one of the key factors affecting the viscosity of post-heating fermented milk, since the heating temperature directly affected the formation of microgels and their aggregation kinetics during the stirring operation and subsequent storage [14]. Stirring gradually disrupted the interaction bonds and structure of fermented milk gels, leading to the formation of microgels [14], while the heating temperature under stirring conditions further affected the size, properties of the microgels, and their interactions in the gel network, thereby affecting the reorganization and aggregation of protein networks during subsequent storage [15]. In the present study, post-heating treatments below 65 °C/25 s promoted the formation of small-sized and medium-sized microgels, leading to the formation of a gel network with more interconnections during reorganization, which in turn increased the sample viscosity, while post-heating treatments above 65 °C/25 s resulted in a decrease in sample viscosity due to the formation of more large-sized particle clusters with weak interactions, forming a heterogeneous and rough gel structure.

#### 3.2.4. Texture Properties

**Figure 6:**
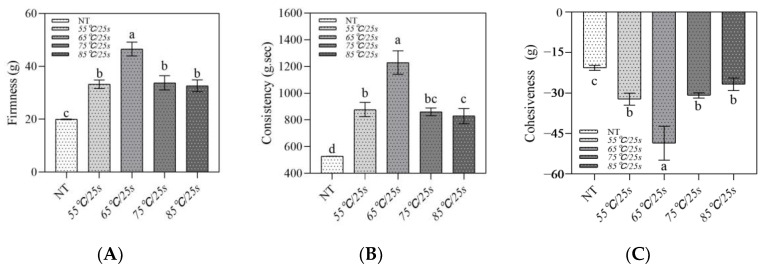
Texture analysis of post-heating stirred fermented milk. Note: The error bars represent the standard error of the means. (**A**–**C**) represent the firmness, consistency, and cohesiveness of the samples, respectively. Values with different letters, in each figure, are significantly different (*p* ≤ 0.05).

Texture is a key aspect affecting consumer acceptance of yogurt. The texture profile analysis results of the samples are shown in Figure 6, and three texture parameters including firmness, consistency, and cohesiveness were obtained. As depicted in Figure 6A–C, the texture parameters of the samples showed a similar trend; that is, each texture parameter increased significantly (*p* ≤ 0.05) as the temperature increased from 55 °C/25 s to 65 °C/25 s, and then decreased significantly (*p* ≤ 0.05) as the temperature increased from 75 °C/25 s to 85 °C/25 s, but no significant difference was found between the 75 °C/25 s and 85 °C/25 s groups (*p* > 0.05). According to Gilbert et al. [24] and Saleh et al. [25], firmness could be used to reflect the structural integrity of yogurt and was related to microstructure dependent on interactions between molecules or particles, while cohesiveness could indicate the degree of deformation of yogurt during testing and was usually discussed in terms of bond strength. Therefore, the bond types, particle sizes, and protein interactions of the samples could contribute to the textural properties [26]. In this paper, post-heating treatments below 65 °C/25 s increased the size of microgels and the number and strength of bonds between microgel clusters, resulting in a compact, homogeneous, more mechanical stress-resistant and rigid gel network, thereby increasing the firmness and consistency of the samples. Also, higher cohesiveness was also associated with a stronger gel structure. However, post-heating treatments above 65 °C/25 s caused excessive aggregation of microgels, forming large irregular and weakly interacting microgel clusters, which resulted in a rough microstructure of the gel network, leading to a decrease in textural features.

### 3.3. Tribology

**Figure 7:**
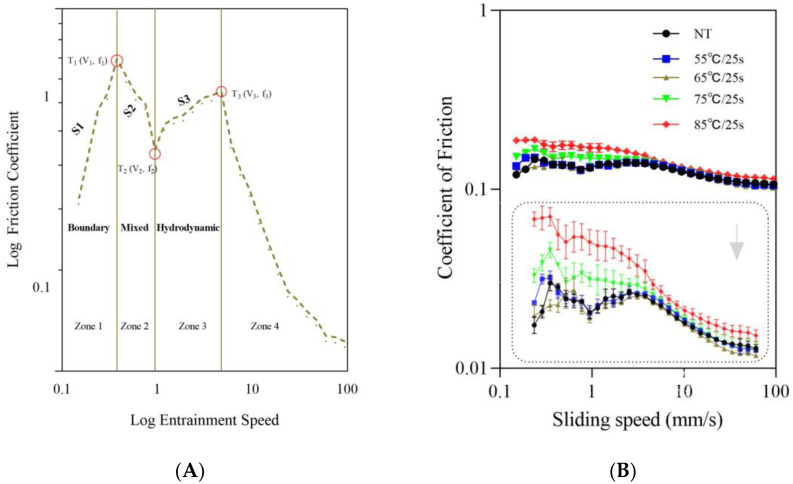
Traditional Stribeck curves (**A**) and friction curves (**B**) of post-heating stirred fermented milk. Note: The error bars represent the standard error of the means (**B**).

As shown in Figure 7B, the lubrication properties of post-heating stirred fermented milk were determined by measuring the coefficient of friction against the sliding speed. According to the typical Stribeck curve (Figure 7A) [27], the average friction curve of the samples can be divided into three regimes: boundary, mixed, and hydrodynamic. In the boundary regime (Zone 1), the friction between two contact surfaces is hardly affected by the sliding speed or sample viscosity, but is related to the sample adsorption capacity and the ability to form a lubrication film between the two surfaces; in the mixed regime (Zone 2), the sample friction decreases with the increasing sliding speed due to the creation of a hydrodynamic film, which is mainly attributed to the sample viscosity that promotes fluid entrainment; in the hydrodynamic regime (Zone 3), as the sliding speed further increases, the hydrodynamic film is fully developed and the two contact surfaces are completely separated, where the friction is mainly influenced by internal friction or sample viscosity and increases with the sliding speed. Additionally, Zone 4 also depicts friction at high speeds when the gel structure disintegrates, and the friction drops [8]. Furthermore, to quantify the friction behavior in each zone, we also defined v_i_ and f_i_ to be the velocity (mm/s) and friction coefficient at the transition point T_i_, respectively, and S_i_ to be the slope in Zone i (assuming a linear relationship between the logarithm of friction and the logarithm of entrained velocity in each zone) [23], where i = (1, 3). The values of these parameters for all fermented milk samples are shown in Table 2.

Combining Figure 7 and Table 2, all samples exhibited a very short boundary regime (Zone 1), and the friction coefficient in this regime increased with the sliding velocity. Generally speaking, at low sliding speeds (<0.24 mm/s, Table 3), the gap between the two contact surfaces was very narrow and only liquid whey could enter the contact zone, so the friction behavior in this zone was dominated by soluble substances and small-dispersed particles in liquid whey, which migrated from the gel matrix [27]. However, for the post-heating samples in this study, the liquid whey may not only contain soluble substances such as conventional whey protein and free fat globules, but also some protein aggregates formed due to post-heating treatment that can enter the contact surfaces; these protein aggregate particles may pack on the surface, impeding their rolling motion [8], and resulting in an increased coefficient of friction. Another noteworthy finding was that in this zone, the coefficients of friction of the samples above 65 °C/25 s were higher than those of the samples below 65 °C/25 s, which was mainly considered to be related to their gel structure and particle size distribution differences. As the gel structure of the former was relatively denser, the amount of whey and the number of particles that could enter the contact surfaces were reduced, while the gel structure of the latter was looser, and the migration of whey and the size and number of particles that could enter the contact surfaces were increased.

As the sliding speed increased, the fluid (in the form of a soft gel) began to be entrained into the contact zone, forming a thin hydrodynamic film that could partly separate the surfaces [27], and thus the friction began to decrease (Zone 2, mixed regime). We observed that samples above 65 °C/25 s had lower v_2_ values and higher absolute values of S_2_ than samples below 65 °C/25 s, which indicated that their lubrication films formed earlier, also implying that at a lower sliding speed, their microgels were easier to disperse and fluid entrainment was easier to form [27].

As the sliding speed increased further, the lubrication film was fully developed, and its thickness increased so that the two contact surfaces were completely separated; the friction behavior of this zone (Zone 3, hydrodynamic regime) was considered to be mainly related to the sample’s viscosity and internal friction characteristics [27]. In this zone, samples above 65 °C/25 s had lower v_3_ values and higher friction coefficients than samples below 65 °C/25 s, indicating that the samples above 65 °C/25 s had a higher friction performance although their lubrication films were developed earlier. Meanwhile, the higher S_3_ values of the samples above 65 °C/25 s also indicated that their friction behaviors developed faster with the sliding speed, while the same S_3_ values among the samples below 65 °C/25 s indicated that their friction behaviors were similar. These tribological changes in this zone could be explained by variations in their viscosity and the amount and size of particles contained in the post-heating samples. Typically, more viscous samples tend to form thicker, possibly more structured films, while thicker polymer layers in turn reduce the total gap height and fluid flow into them [23]. Therefore, in this study, the samples with lower viscosity above 65 °C/25 s flowed more fluids between the contact surfaces, and these fluids contained more large-sized particles, compared to samples with lower viscosity.

When the sliding speed increased to a certain extent, the gel structure was destroyed and the friction of the samples showed a decreasing trend (Zone 4); Table 2 showed that samples above 65 °C/25 s required lower sliding speeds to break the gel networks than samples below 65 °C/25 s (0.60 vs. 2.37 mm/s), which was consistent with the rheological results (Section 3.2.1). At the same time, the samples above 65 °C/25 s also had a higher friction performance in this zone than the samples below 65 °C/25 s.

### 3.4. Sensory Analysis

Quantitative descriptive testing (*n* = 10) and consumer preference testing (*n* = 50) were used to analyze the sensory attributes of post-heating stirred fermented milk, as shown in Table 3. No significant differences (*p* > 0.05) were observed in the thickness, stickiness, and residual coating among the samples below 65 °C/25 s, while the samples above 65 °C/25 s showed higher residual coating scores, and lower thickness and stickiness scores than the samples below 65 °C/25 s. A decrease in the thickness of the samples above 65 °C/25 s was in agreement with their textural, rheological, and tribological behaviors and lower v_3_ values in the friction curves observed in Section 3.2 and Section 3.3, which were attributed to the formation of large-sized microgel clusters and their weakened interactions, which made the product less likely to stick to oral surfaces. Meanwhile, a decrease in cohesiveness inside the samples above 65 °C/25 s could cause the residue of large-sized particulate matter after swallowing, resulting in the undesirable sensory attribute of residual coating [27]. This, in turn, may also contribute to the samples being less smooth and grainier [27]. Graininess and smoothness were the most affected sensory attributes of post-heating samples, and their similar evaluation results were obtained by trained panelists and consumers. Briefly, there was no significant difference (*p* > 0.05) in the smoothness and graininess scores of samples below 55 °C/25 s, while samples above 55 °C/25 s exhibited significantly reduced smoothness (*p* ≤ 0.05) and significantly increased graininess (*p* ≤ 0.05). These results could be explained by their particle size variations (Section 3.1) and friction behaviors (Section 3.3).

### 3.5. Correlation between the Instrumental Data and Sensory Properties of Post-Heating Stirred Fermented Milk

**Figure 8:**
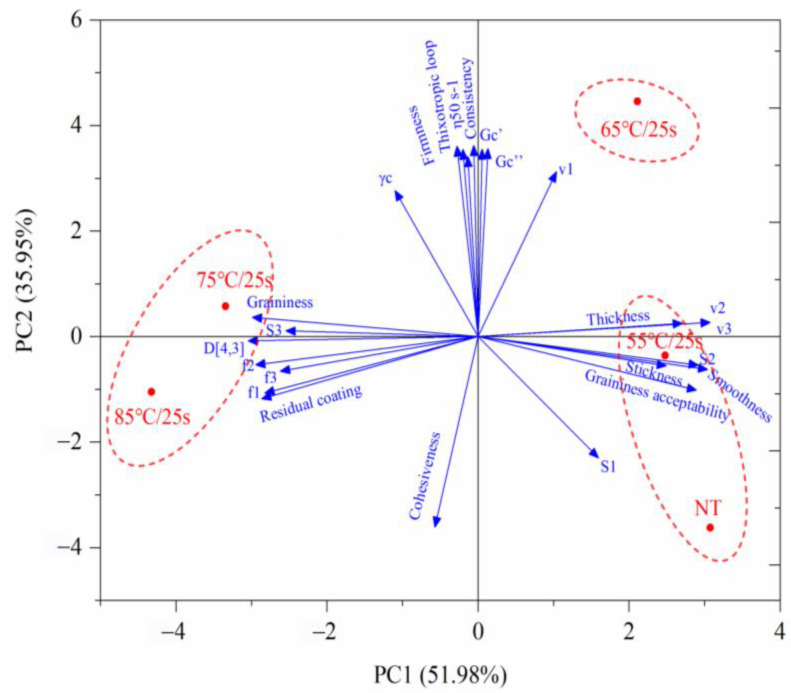
PCA-biplot of instrumental data and sensory attributes of post-heating fermented milk.

The PCA was applied to correlate sensory properties and instrumental data including rheology, texture, tribology, and particle size D_[4,3]_. As shown in Figure 8, the corresponding loading plots of the first two major components, where the first two factors with eigenvalues greater than 1 explained 87.93% of the total variance, showed that the first factor accounted for 51.98%, and the second factor accounted for 35.95%. Also, the correlation coefficients between the two major components and each indicator are also presented in Table 4.

As shown in Figure 8, except for S_1_ and v_1_, the tribological indicators and sensory attributes were mainly explained by PC1, among which, v_2_ (0.99), v_3_ (0.99), S_2_ (0.94), stickiness (0.80), and smoothness (0.98) were negatively correlated with PC1, while D_[4,3]_ (−0.98), graininess 1 (−0.96), residual coating (−0.92), f_2_ (−0.95), f_3_ (−0.84), and S_3_ (−0.82) were positively correlated with PC1. Since v_2_ and v_3_ corresponded with the entrainment speed at the transition points between zones 2 and 3 and zones 3 and 4, respectively, they characterized the speeds at which the hydrodynamic fluid film started to entrain and develop fully between the two contact surfaces, and their values could be associated with sensory properties relating to bulk fluid properties, such as smoothness [27]. As mentioned in Section 3.3, slopes S_2_ and S_3_ corresponded to the entrainment of fluid gel in the form of (thin) and (thick) lubrication film, respectively. Consequently, S_2_ could be related to the oral stickiness associated with thin film properties, and the higher the S_2_ value, the greater the ability of the lubrication film to entrain and adsorb onto the tape surface [27], which meant that the samples were more likely to stick to the oral surface; S_3_ could be related to the residual coating associated with thick film properties, and the higher the S_3_ value, the thicker the lubrication film containing more large-sized particles, and the more residual after swallowing. Furthermore, S_2_ and S_3_ also appeared to be thickness-dependent, suggesting that the thickness of the fluid not only affected its frictional behavior at high speeds (large gap), but also affected the friction of the lubrication film at low speeds (narrow gap). Finally, according to the explanation ratio of PC1 and PC2, the five treatment groups were roughly clustered into three categories: NT group and 55 °C/25 s group, 75 °C/25 s group and 85 °C/25 s group, and 65 °C/25 s group.

### 3.6. Regression Analysis of Graininess and Graininess Acceptability of Post-Heating Fermented Milk

Based on the PCA analysis and empirical relationships reported in the earlier literature [28,29], the two critical sensory properties (graininess and graininess acceptability) of the fermented milk samples were regressed against the particle size D_[4,3]_ and tribological parameters including S_2_ and S_3_. Different particle sizes should result in different perceptions of graininess [29]. Because the empirical relationships were not linear, the measurement logarithm was used [29]. Meanwhile, since the S_2_ and S_3_ values were related to the ability of the lubrication film to entrain and adsorb onto the tape surface, its wettability was similar to that of the human tongue [30], so they were also included in the graininess acceptability model together with D_[4,3]_. The corresponding regression equations were as follows:

Log10 [Graininess] = 0.586 × Log10 [(D_[4,3]_)] − 0.231, R^2^ = 0.94, *p* < 0.05 (1)

Log10 [Graininess acceptability] = 0.133 × Log10 [(D_[4,3]_)] + 5.377S2 + 7.748S_3_ + 0.716, R^2^ = 0.93, *p* < 0.05 (2)

**Figure 9:**
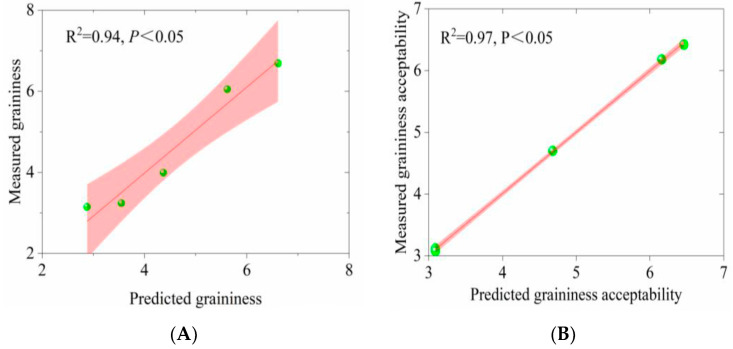
Multiple linear regression plots for graininess (**A**) and graininess acceptability (**B**) measured compared with those predicted by means of particle size (D_[4,3]_) and tribologically derived parameters as a function of confidence interval limits (*p* < 0.05).

As shown in Figure 9, the goodness-of-fit statistics for the graininess model showed promising predictive values with R^2^ = 0.94 (Figure 9A), and a similar fit (R^2^ = 0.97, Figure 9B) was found for the graininess acceptability model.

It needs to be stated that, although a wide variety of different post-heating fermented milk systems were investigated within this work, the validity of the relationship between instrumental and sensory data presented in the obtained regression models remains to be examined in other fermented milk systems.

## 4. Conclusions

This study demonstrated that the aggregation degree and aggregate size of the microgels driven by post-heating temperatures determined the textural and sensory attributes of the fermented milk. Post-heating treatments below 65 °C/25 s appeared to favor the textural properties of stirred fermented milk, as the improved gel structural properties brought about by moderate aggregation (~15 μm–~21 μm) offset the undesirable sensory defects with a focus on graininess caused by slightly increased aggregate sizes. However, post-heating treatments above 65 °C/25 s tended to produce undesirable sensory (reduced stickiness and smoothness, increased graininess, and residual coating) and textural (decreased viscosity, firmness, and consistency) defects due to a combination of increased aggregate size and deteriorated gel structural properties caused by excessive aggregation (~46 μm–~63 μm). These findings may provide an understanding of the formation of textural and sensory defects in ambient fermented milk products and a perspective on improving these defects by controlling the aggregation process. To the best of our knowledge, this is the first study to investigate the changes in the textural properties of stirred fermented milk induced by different post-heating temperatures (55–85 °C, 25 s) and their contributions to the sensory attributes. In the future, further consideration will be given to expanding the post-heating temperature range (even to 95 °C) or increasing the number and variability of samples in independent experiments, with the aim of expanding the applicability of this study and strengthening the robustness of these existing findings. Simultaneously, long-term stability and several key quality aspects such as flavor, texture, rheology, and syneresis during room temperature storage are also considered for further studies aimed at evaluating the effectiveness of these post-heating treatment conditions in their application.

## Figures and Tables

**Table 1:** Particle size distribution parameters of post-heating stirred fermented milk.

	D_[10]_ (μm)	D_[50]_ (μm)	D_[90]_ (μm)	D_[4,3]_ (μm)
NT	7.30 ± 0.13 ^e^	13.63 ± 0.21 ^e^	24.98 ± 0.52 ^e^	15.02 ± 0.20 ^e^
55 °C/25 s	8.83 ± 0.01 ^d^	17.72 ± 0.04 ^d^	38.65 ± 0.30 ^d^	21.27 ± 0.16 ^d^
65 °C/25 s	10.52 ± 0.04 ^c^	25.04 ± 0.18 ^c^	61.20 ± 0.94 ^c^	31.08 ± 0.36 ^c^
75 °C/25 s	14.73 ± 0.15 ^b^	41.05 ± 0.58 ^b^	87.30 ± 1.46 ^b^	46.60 ± 0.58 ^b^
85 °C/25 s	16.40 ± 0.32 ^a^	55.87 ± 1.60 ^a^	122.17 ± 3.31 ^a^	63.23 ± 1.82 ^a^

Data represent mean values ± standard deviations. Different letters in the same column represent significant differences (*p* ≤ 0.05).

**Table 2:** Parameters obtained from tribology model for post-heating stirred fermented milk.

	Tribological Parameters								
	v_1_	f_1_	v_2_	f_2_	v_3_	f_3_	S_1_	S_2_	S_3_
NT	0.24	0.15 ± 0.01	0.75	0.13 ± 0.00	2.37	0.14 ± 0.00	0.31 ± 0.08	−0.03 ± 0.01	0.01 ± 0.00
55 °C/25 s	0.24	0.15 ± 0.01	0.75	0.13 ± 0.00	2.37	0.14 ± 0.00	0.17 ± 0.04	−0.03 ± 0.01	0.01 ± 0.00
65 °C/25 s	0.47	0.14 ± 0.00	0.75	0.13 ± 0.01	2.37	0.14 ± 0.00	0.07 ± 0.01	−0.06 ± 0.02	0.01 ± 0.00
75 °C/25 s	0.24	0.17 ± 0.00	0.38	0.15 ± 0.00	0.60	0.15 ± 0.00	0.18 ± 0.05	−0.17 ± 0.03	0.06 ± 0.01
85 °C/25 s	0.24	0.19 ± 0.01	0.38	0.17 ± 0.01	0.60	0.18 ± 0.01	0.03 ± 0.01	−0.13 ± 0.03	0.03 ± 0.01

Note: Data represent mean values ± standard deviations. v_1_, v_2_, and v_3_ represented the transition point velocities (mm/s) corresponding to Zone 1, Zone 2, and Zone 3 presented in Figure 7A, respectively; f_1_, f_2_, and f_3_ represented the friction coefficients corresponding to v_1_, v_2_, and v_3_, respectively; S_1_, S_2_, and S_3_ represented the slopes corresponding to Zone 1, Zone 2, and Zone 3 presented in Figure 7A, respectively.

**Table 3:** Sensory evaluation of post-heating stirred fermented milk.

	Quantitative Descriptive Analysis (QDA)					Consumer Preference Analysis (CPA)
	Trained Panelists (*n* = 10)					Consumers (*n* = 50)
Groups	Thickness	Smoothness	Graininess	Stickiness	Residual Coating	Graininess Acceptability
NT	6.14 ± 0.17 ^a^	7.35 ± 0.71 ^a^	3.15 ± 0.58 ^d^	5.59 ± 0.18 ^a^	4.50 ± 0.33 ^b^	6.18 ± 1.39 ^a^
55 °C/25 s	5.93 ± 0.22 ^ab^	7.03 ± 0.33 ^a^	3.24 ± 0.40 ^d^	5.87 ± 0.10 ^a^	4.69 ± 0.28 ^ab^	6.42 ± 1.43 ^a^
65 °C/25 s	6.15 ± 0.23 ^a^	5.69 ± 0.58 ^b^	3.99 ± 0.45 ^c^	5.50 ± 0.13 ^a^	4.36 ± 0.29 ^b^	4.70 ± 1.48 ^b^
75 °C/25 s	5.57 ± 0.33 ^b^	3.18 ± 0.32 ^c^	6.05 ± 0.31 ^b^	5.10 ± 0.54 ^b^	4.96 ± 0.29 ^a^	3.12 ± 1.56 ^c^
85 °C/25 s	5.75 ± 0.31 ^ab^	2.39 ± 0.33 ^d^	6.69 ± 0.22 ^a^	5.33 ± 0.28 ^b^	5.15 ± 0.33 ^a^	3.08 ± 1.10 ^c^

Note: Data represent mean values ± standard deviations. Different letters in the same column represent significant differences (*p* ≤ 0.05).

**Table 4:** Correlation coefficients of instrumental data and sensory attributes with the two major components.

Attributes	Axis1	Axis2
Instrumental		
Thixotropic loop	−0.07	0.96
η_50 s-1_	−0.04	0.93
γc	−0.35	0.75
Gc′	0.02	0.96
Gc″	0.04	0.97
Firmness	−0.09	0.98
Consistency	−0.02	0.98
Cohesiveness	−0.18	−0.98
D_[4,3]_	−0.98	−0.02
v1	0.33	0.85
f1	−0.91	−0.29
v2	0.99	0.07
f2	−0.95	−0.15
v3	0.99	0.07
f3	−0.84	−0.18
S1	0.51	−0.62
S2	0.94	−0.15
S3	−0.82	0.03
Sensory		
Thickness	0.87	0.07
Smoothness	0.98	−0.17
Graininess	−0.96	0.10
Stickiness	0.80	−0.15
Residual coating	−0.92	−0.32
Graininess acceptability	0.93	−0.28

## Data Availability

The data used to support the findings of this study can be made available by the corresponding author upon request.
